# Protein aggregation as a bistable switch in bacterial cell fate: from adaptive dormancy to cytotoxic death

**DOI:** 10.3389/fmicb.2026.1929318

**Published:** 2026-08-27

**Authors:** Yuejuan Nong, Weijie Wang, Weiwei Zhu

**Affiliations:** 1 State Key Laboratory of Vaccines for Infectious Diseases, Xiang-An Biomedicine Laboratory, National Innovation Platform for Industry-Education Integration in Vaccine Research, Department of Laboratory Medicine, School of Public Health, Xiamen University, Xiamen, China; 2 Research Center for Clinical Medicine, The First Affiliated Hospital of Kunming Medical University, Kunming, China; 3 Yunnan Province Clinical Research Center for Laboratory Medicine, Kunming, China

**Keywords:** bacterial aging, bacterial dormancy, bacterial proteostasis, cell death, protein aggregation, stress response

## Abstract

Protein aggregation has traditionally been considered a hallmark of proteostasis disruption and cellular dysfunction. However, recent studies have revealed that bacterial protein aggregation is not merely a passive consequence of stress-induced damage but may represent a dynamic component of cellular adaptation. Under adverse conditions, reversible protein condensation and aggregation have been associated with bacterial dormancy, persistence, and the viable but non-culturable (VBNC) state, whereas excessive and irreversible aggregation may contribute to loss of cellular function and bacterial death. Nevertheless, whether protein aggregation serves as a primary determinant of bacterial cell fate or reflects a consequence of broader physiological changes remains an important unresolved question. In this review, we summarize current advances in understanding bacterial protein aggregation, including the roles of liquid–liquid phase separation (LLPS), protein quality control systems, ATP-dependent proteostasis regulation, and aggregate maturation. We discuss how stress-induced alterations in proteostasis networks influence bacterial survival, aging, persistence, and antibiotic tolerance. Available evidence supports a working model whereby the physicochemical properties of protein aggregates may dictate ultimate bacterial cell fate. Nevertheless, the causal link between condensate phase behavior, aggregate material characteristics, and cell fate decisions remains to be thoroughly validated, even though emerging technologies including live single-molecule cell imaging and quantitative analytical tools have greatly advanced our mechanistic understanding of the dynamic progression of bacterial protein aggregation. Finally, we discuss the therapeutic potential and challenges of targeting bacterial proteostasis and aggregation pathways to combat persistent and multidrug-resistant pathogens. A deeper mechanistic understanding of how protein aggregation balances adaptation and toxicity may reveal new vulnerabilities within bacterial survival strategies.

## 1 Introduction

Proteostasis refers to the finely tuned dynamic equilibrium that cells maintain throughout the lifespan of proteins, encompassing their synthesis, proper folding, functional performance, and ultimate degradation. It is an essential prerequisite for normal cellular activities (Laskowska et al., 2019; Khodaparast et al., 2021). Bacteria endure relentless survival pressures in complex and fluctuating natural microecological niches, as well as during clinical anti-infective interventions. For example, drastic temperature shifts, oxidative stress, nutrient limitation, and antibiotic exposure can all severely perturb proteostasis in bacterial cells (Kwiatkowska et al., 2008; Laskowska et al., 2019). Bacteria have developed a protein quality control system comprising molecular chaperones and proteases to maintain normal physiological functions (Diaz Arenas et al., 2025). Severe environmental stress disrupts proteostasis, causing massive misfolding that irreversibly exposes internal hydrophobic domains and results in intracellular protein aggregates (Bollen et al., 2021).

Extensive protein aggregation has long been regarded as a pathological marker of full proteostasis failure (Schramm et al., 2020). Early studies highlight the toxic effects of protein aggregates: non-specific aggregation competes for the protein quality-control machinery and cellular energy, disrupts macromolecular complex assembly, and ultimately promotes bacterial senescence and irreversible cell death (Olzscha et al., 2011; Bednarska et al., 2013). Recent breakthroughs in single-cell time-lapse imaging and microfluidics have led to an accumulating body of studies demonstrating that bacterial protein aggregation has dual biological functions, with a delicate dynamic boundary between toxic pathology and physiological adaptation (Bednarska et al., 2013; Schramm et al., 2020). Through evolution, bacteria have transformed this potentially lethal damage into a proactive survival strategy (Bollen et al., 2021). Under adverse conditions, aggregates may function as a highly regulated physiological apparatus rather than as passive pathological products, and as a factor to promote bacterial dormancy (Pu et al., 2019; Bollen et al., 2021).

Overall, stress-induced proteostasis perturbation is not merely a lethal event, but an important physiological turning point that determines whether bacteria undergo toxic cell death or enter adaptive dormancy (Bednarska et al., 2013; Dewachter et al., 2021). Antibiotic resistance has emerged as a major global public health challenge. Deep dormancy mediated by protein aggregates is a key mechanism that enables bacteria to evade antibiotic killing and, consequently, cause recurrent infections in clinical settings (Pu et al., 2019; Bollen et al., 2021; Khodaparast et al., 2021). Accordingly, this review aims to summarize recent advances in research on the disruption of proteostasis and cell fate determination in bacteria.

## 2 The formation of protein aggregates in bacteria

Two primary drivers govern protein aggregation in bacteria. Endogenous perturbation arises from intracellular crowding, translational errors, and high basal metabolic activity. The other category comprises acute heat shock and additional lethal exogenous insults (Schramm et al., 2020; Bollen et al., 2021). Regardless of the trigger, the assembly and development of abnormally exposed polypeptide chains represent a sequential process from focal damage to the breakdown of overall proteostasis (Mogk et al., 2011; Hipp et al., 2019; Schramm et al., 2020).

### 2.1 Endogenous perturbation and proteostatic burden

Bacterial proteostasis exists in a highly dynamic, precarious steady state even under stress-free growth conditions (Schramm et al., 2020; Bollen et al., 2021). This basal proteostatic burden fundamentally arises from endogenous perturbations that accompany routine physiological processes (Khodaparast et al., 2021).

#### 2.1.1 Effect of macromolecular crowding

The bacterial cytoplasm represents a highly crowded space containing abundant proteins, nucleic acids, lipids, and supramolecular complexes (Konopka et al., 2006). Proteins and rRNA dominate the intracellular macromolecular pool and are primarily responsible for physical crowding (Poolman, 2023). The dense physiological environment drastically limits the free space for macromolecular movement. Steric occupancy by numerous crowding agents reduces the effective cellular volume, a phenomenon known thermodynamically as the excluded volume effect (Minton, 2004; Poolman, 2023). It diminishes the number of free water molecules within the system, driving macromolecules to assume maximally compact conformations and reduce spatial occupancy (Gimón et al., 2026). While this helps stabilize native protein folding to some extent, it accelerates the non-specific binding of abnormal intermediates as proteins undergo conformational fluctuations under environmental stress. Exposed hydrophobic interfaces drive damaged polypeptides to oligomerize and aggregate, thereby decreasing the total excluded volume and free energy of the system (Minton, 2004; Schramm et al., 2020). The low free-energy state confers high stability on aggregates, making aggregation highly irreversible.

In addition, intracellular macromolecular crowding imposes spatial confinement and markedly slows the lateral diffusion of soluble proteins (Konopka et al., 2006). Diffusional hindrance raises local macromolecule concentrations dramatically. Retained misfolded chains collide and form aberrant cross-links far more frequently (Konopka et al., 2006; Schramm et al., 2020). Together, thermodynamic propensity for cross-linking and kinetic diffusion limitations push subtle folding perturbations beyond the reversible repair threshold, culminating in massive protein aggregation (McKeever et al., 2026).

#### 2.1.2 Translational errors in proteins

In bacteria, genetic information flows through DNA replication, transcription and translation, and interference with any step can cause protein translational errors (Schwartz and Pan, 2017). Whole-proteome mass spectrometry analyses show the average amino acid misincorporation rate in *Escherichia coli* is ∼10^–3^ (one error per 1000 residues). Combined effects of codon features, protein properties and physiological conditions cause the *in vivo* error rate to fluctuate by over an order of magnitude (Mordret et al., 2019). Cells sustain folding homeostasis via codon usage bias, favoring synonymous codons matched with abundant cognate tRNAs to lower misincorporation (Sun and Zhang, 2022). According to a translational error model developed by Landerer et al., 20%–23% of newly synthesized proteins contain at least one misincorporated amino acid (Landerer et al., 2024). Although targeted mistranslation is triggered for adaptation under stress (Netzer et al., 2009; Wiltrout et al., 2012; Schwartz and Pan, 2016; Schwartz et al., 2016), unregulated translational errors typically perturb native protein conformations, induce inactivation and misfolding, and promote protein aggregation (Schwartz and Pan, 2017).

### 2.2 Exogenous stress-induced proteostasis disruption

While bacterial proteostasis networks possess strong adaptive capacity to maintain intracellular homeostasis (Winkler et al., 2010), intense exogenous stress rapidly disrupts the thermodynamic and biochemical equilibrium of cellular proteostasis, far beyond the basal folding load of endogenous metabolism, leading to failure of protein quality control machinery (Balchin et al., 2016; Schramm et al., 2020).

#### 2.2.1 Heat and osmotic stress

Temperature acts as a central determinant governing protein structure and proteostasis. Upon abrupt environmental temperature elevation (namely heat shock) in bacteria, non-covalent interactions sustaining higher-order protein conformations are rapidly disrupted, triggering protein unfolding and exposing hydrophobic residues originally buried in the molecular interior (Anfinsen and Scheraga, 1975). These aberrantly exposed polypeptide chains are predominantly driven by non-specific hydrophobic forces, coupled with sequence-specific cross-linking via β-sheet interactions. This cascade subsequently induces extensive co-aggregation and ultimately yields poorly soluble amorphous aggregates (Mogk et al., 1999; Balchin et al., 2016; Pu et al., 2019). In *E. coli*, heat stress drives aggregation of up to 15%–25% of soluble cytoplasmic proteins, exerting particularly severe detrimental effects on proteins with molecular masses ≥ 90 kDa (Mogk et al., 1999).

Osmotic stress triggers acute cellular dehydration, elevated intracellular ionic strength, cell shrinkage, and macromolecular crowding (Burg et al., 2007; Choe and Strange, 2008). Studies have demonstrated that osmotic stress-induced cell shrinkage also constitutes an important mechanism driving aberrant protein aggregation (Krokowski et al., 2022; Burkewitz et al., 2011; Miermont et al., 2013). Furthermore, instantaneous efflux of water from bacterial cells directly strips the protective hydration shell that maintains the native conformation of polypeptide chains, exposing the hydrophobic protein cores and consequently promoting abnormal protein aggregation (Ignatova and Gierasch, 2006; Stadmiller et al., 2017). Under hyperosmotic stress, essential macromolecules with highly dynamic conformations–such as RNA-binding proteins–are prone to conformational perturbation, forming degradation-resistant co-aggregation networks (Zaepfel and Rothstein, 2021; Gao et al., 2022). The sharply elevated burden of misfolded proteins overwhelms the protein quality control machinery, ultimately resulting in disrupted proteostasis.

#### 2.2.2 Oxidative damage and antibiotic exposure

Beyond physical stressors, oxidative stress disrupts the higher-order structures of proteins, thereby impairing their activity, stability, and solubility (Dahl et al., 2015). In bacteria, intricate cellular redox states and the corresponding signaling networks can modulate, or even directly initiate and propagate, protein aggregation (van Dam and Dansen, 2020). Sulfur-containing amino acid residues within polypeptide chains are highly susceptible to oxidative stress (Dahl et al., 2015; Ezraty et al., 2017). Under mild oxidative stress, reactive oxygen species (ROS) specifically react with sulfur-bearing residues to form reversible disulfide bonds, a reversible post-translational modification that serves as an adaptive cellular redox signal (D’Autréaux and Toledano, 2007; Winterbourn and Hampton, 2008; Schieber and Chandel, 2014). Early-stage protein aggregates arising from local conformational unfolding are largely sustained by weak non-covalent interactions (Morris et al., 2008; Chiti and Dobson, 2017). Heat shock proteins such as CnoX selectively bind unfolded substrate proteins and block irreversible carbonylation damage or permanent aggregation via formation of mixed disulfide complexes (Goemans et al., 2018). Upon removal of oxidative stress and restoration of intracellular reducing potential, these nascent aggregates can be disaggregated and refolded with the aid of antioxidant systems including thioredoxins and ATP-dependent molecular chaperones (Winter et al., 2005; Ezraty et al., 2017).

Nevertheless, exposure of *E. coli* to lethal doses of ROS derived from external environments or the host immune system disturbs intracellular redox homeostasis. This perturbation further triggers the destabilization of iron-sulfur clusters, which release free ferrous ions to catalyze the Fenton reaction and generate abundant hydroxyl radicals (Imlay, 2013; Dahl et al., 2015). Severe oxidative stress not only induces oxidation of guanine nucleotides but also elicits irreversible protein carbonylation, which severely disrupts protein architecture (Stadtman and Levine, 2003; Foti et al., 2012; Ezraty et al., 2017). Collectively, ROS exert dose-dependent effects on proteostasis, shifting from reversible signaling functions to irreversible destructive damage as stress intensity rises.

Furthermore, ROS are recognized as secondary lethal mediators for diverse antibiotics (Kohanski et al., 2007, 2010; Belenky et al., 2015). While bactericidal antibiotics inhibit their molecular targets, they simultaneously perturb central metabolism and trigger a massive surge in secondary hydroxyl radicals. Excess ROS mediate oxidative modification of intracellular guanine nucleotides, elevating the error rates of transcription and translation and accelerating aberrant protein aggregation at its source (Foti et al., 2012). Continuous accumulation of aggregates in turn disrupts intracellular redox homeostasis and provokes secondary ROS buildup (van Dam and Dansen, 2020), forming a vicious cycle of “translation errors-oxidative damage-aggravated aggregation.” This positive feedback amplifies localized folding defects and sustains progressive protein aggregation.

Specifically, aminoglycoside antibiotics target bacterial ribosomes and drastically compromise translational fidelity, generating abundant polypeptide chains with sequence mismatches and folding defects intracellularly, thereby disrupting bacterial proteostasis at the protein synthesis stage (Kohanski et al., 2008). Further proteome-wide investigations have revealed that aminoglycosides do not merely induce sporadic single amino acid misincorporations. By inhibiting ribosomal translocation, these drugs trigger clustered translational errors featuring consecutive amino acid substitutions on nascent polypeptide chains. This phenomenon constitutes the primary mechanism underlying severe proteotoxic stress in bacteria exposed to low concentrations of aminoglycosides (Wohlgemuth et al., 2021).

Upon exposure to antibiotics and the concomitant oxidative stress, bacteria deploy robust survival strategies. Deep dormancy phenotypes mediated by protein aggregation can reduce bacterial susceptibility to ofloxacin and amikacin (Dewachter et al., 2021). Under stress conditions, *E. coli* forms insoluble aggregated structures termed “regrowth-delay body” that sequester essential metabolic enzymes including FtsZ, enabling bacterial survival against ampicillin or ofloxacin (Yu et al., 2019). Additional studies have demonstrated that trimethylamine N-oxide (TMAO) acts as a chemical chaperone analog. It likely stabilizes intracellular protein conformations and protects proteins from denaturation and damage, thereby diminishing the susceptibility of *E. coli* to a broad spectrum of antibiotics (Qiao et al., 2022). These findings underscore the key role of proteostasis in the bacterial response to antibiotic pressure.

### 2.3 Protein quality control and ATP depletion

Intracellular ATP levels are indispensable for the maintenance of proteostasis. For one thing, protein synthesis is inherently an energy-consuming process, and ATP at physiological concentrations functions as a biological hydrotrope (Patel et al., 2017). Owing to its amphipathic molecular architecture consisting of hydrophobic aromatic rings and negatively charged triphosphate moieties, ATP effectively reduces the surface tension of hydrophobic interfaces. This property preserves protein solubility within the crowded cytoplasmic milieu and suppresses macromolecular aggregation (Rice and Rosen, 2017). For another, bacteria rely on the protein quality control network–composed of molecular chaperones such as the DnaK-ClpB system and AAA+ proteases–to refold or eliminate aberrant proteins (Diaz Arenas et al., 2025), and the proper operation of this machinery strictly relies on energy derived from ATP hydrolysis.

Correspondingly, diminished ATP pools disrupt cellular proteostasis via two distinct mechanisms (Pu et al., 2019). On the one hand, ATP depletion directly impairs its capacity as a biological hydrotrope (Rice and Rosen, 2017; Pu et al., 2019). On the other hand, energy deficiency halts the catalytic cycles of the PQC system and paralyzes chaperone-mediated refolding pathways (Flanagan and Bewley, 2002; Diaz Arenas et al., 2025). Meanwhile, deprived of ATP hydrolysis energy, the unfoldase domains of AAA+ proteases lose the mechanical driving force required to remodel damaged polypeptides, stalling both refolding and clearance routes for misfolded proteins (Sauer et al., 2022; Hoskins et al., 2025). Collectively, sufficient intracellular ATP availability serves as an essential prerequisite for cells to sustain intact proteostasis.

When bacteria undergo metabolic stress such as the stationary phase triggered by nutrient deprivation, their metabolic activity is repressed, resulting in a sharp drop in intracellular ATP concentrations. Single-cell and proteomic analyses have further validated that ATP depletion drives the formation of intracellular protein aggregates (Pu et al., 2019). Severe oxidative damage can also induce drastic ATP exhaustion, which in turn causes unfolding and functional inactivation of core ATP-dependent chaperones including DnaK (Winter et al., 2005).

Conversely, bacteria modulate intracellular ATP pools to sustain proteostasis under stress. Njenga et al. systematically elucidated a homeostatic regulatory mechanism: under environmental pressure, bacteria remodel ribosome biogenesis and downregulate global translational rates to dampen the activity of translational machineries. This strategy reduces cellular energy expenditure and concurrently prevents massive production of misfolded polypeptides (Njenga et al., 2023). Meanwhile, Santra et al. constructed a kinetic model of the *E. coli* proteostasis network and revealed that cells selectively sort substrate proteins via chaperone discrimination based primarily on the thermodynamic stability and kinetic accessibility of misfolded conformers. This work demonstrated that bacteria maintain proteostasis with high energy efficiency: the highly energy-consuming GroE chaperone system is exclusively allocated to repair severely damaged proteins that are most refractory to refolding (Santra et al., 2017).

Additionally, cells can accumulate large quantities of inorganic polyphosphate (polyP) to act as temporary ATP-independent scaffolds for compensatory protective responses (Dahl et al., 2015). Nevertheless, prolonged oxidative stress ultimately disrupts the entire stress defense network irreversibly, driving polypeptide chains to assemble into insoluble amorphous aggregates.

## 3 Liquid–liquid phase separation driven remodeling of proteostasis

Under extreme stress, damaged polypeptide chains do not directly and randomly assemble into irreversible solid aggregates. An emerging view posits that proteostasis is remodeled via liquid–liquid phase separation (LLPS), a tightly regulated thermodynamic process (Alberti and Hyman, 2021; Kuczyńska-Wiśnik et al., 2023). Classical biochemical frameworks fail to explain how damaged proteins rapidly assemble into highly ordered defensive or regulatory networks. The introduction of the LLPS paradigm fills this gap in our understanding of the underlying microscale mechanisms. Nevertheless, it should be noted that much of the evidence supporting LLPS as an initiating step of protein aggregation during bacterial stress responses primarily originates from *in vitro* experiments or correlation analyses. Its prevalence and causal significance in the native cellular environment remain to be further verified (Pei et al., 2025; Jin et al., 2021; Kuczyńska-Wiśnik et al., 2023).

Liquid–liquid phase separation-related models suggest that upon exposure to stress in bacteria, the original thermodynamic equilibrium is disrupted, which may lower the energy barrier for spatial self-assembly of proteins. This drives a large pool of structurally sensitive macromolecules rich in intrinsically disordered regions (IDRs) or multivalent binding domains to exceed their solubility thresholds (Alberti et al., 2019; Azaldegui et al., 2021). Polypeptide chains crosslink through transient, highly dynamic multivalent weak interactions and undergo spontaneous LLPS, rapidly assembling into membrane-less biomolecular condensates (Wei et al., 2020). Combining cutting-edge live single-cell imaging and chemical biological tracing techniques, researchers have discovered that assemblies formed by bacteria at the early stage of stress are not static amorphous solid precipitates; instead, they exhibit highly dynamic liquid-like physical properties (Wolstenholme et al., 2020; Farrag et al., 2025). Phase separation is not restricted to soluble macromolecules. Under specific physical stress such as hyperosmotic stress, integral membrane proteins (e.g., lactose permease LacY) can also undergo intramembrane phase separation and condensation mediated by hydrophobic interactions (Linnik et al., 2026).

Existing studies suggest that LLPS not only serves as the physical initiation step for aberrant protein aggregation in bacteria under extreme stress, but may also establish a multi-layered defensive and regulatory barrier for cells at the critical threshold of proteostasis perturbation by constructing highly fluid and reversible liquid-phase networks. Liquid condensates function as transient protective microcompartments that selectively sequester essential macromolecules such as mRNA to shield them from nuclease-mediated degradation (Pei et al., 2025). Meanwhile, phase-separated integral membrane proteins (e.g., LacY) retain or even augment their substrate transport activity upon hyperosmotic stress (Linnik et al., 2026). In addition, LLPS is thought to be deeply involved in the spatiotemporally precise regulation of the bacterial cell cycle. For instance, core cell division proteins (including septum-localized FtsZ) and chromosome segregation regulators (such as MatP) self-assemble into highly dynamic biomolecular condensates, which act as molecular buffering reservoirs and spatial organizing hubs throughout the division cycle (Zorrilla et al., 2021; Barros-Medina et al., 2025).

## 4 Protein aggregation and bacterial cell fate

As outlined above, the phase transition of bacterial protein aggregates acts as a key regulatory hub governing bacterial cell fate, steering cells toward two distinct biological outcomes. On the one hand, the intrinsic cytotoxicity of protein aggregates non-specifically depletes chaperone networks and impedes the assembly of macromolecular complexes, thereby accelerating cellular senescence and irreversible cell death (Lindner et al., 2008; Schramm et al., 2020). On the other hand, bacteria have evolved over long-term to remodel potentially lethal damage into proactive defensive strategies (Pu et al., 2019; Bollen et al., 2021).

### 4.1 Dynamic protein aggregation-mediated metabolic arrest and deep dormancy

Current studies support a model wherein protein aggregation constitutes a dynamic and gradual phase-transition process, which markedly improves bacterial survival under adverse conditions such as antibiotic exposure by driving cells into graded dormant states (Bollen et al., 2021; Sniezek et al., 2025). Studies have demonstrated that when bacteria enter stationary phase or confront environmental stress, intracellular proteostasis is remodeled, leading to progressive accumulation of macromolecular aggregates. These aggregates are thought to sequester indispensable proteins involved in transcription, translation, central metabolism and cell division, triggering systemic ATP depletion (Leszczynska et al., 2013; Pu et al., 2019; Bollen et al., 2021). In *E. coli*, progressive aggregation has been observed to drive the transition from a weakly dormant persister state characterized by early liquid condensates to a deep viable but non-culturable (VBNC) state marked by mature solid aggregates. This supports the hypothesis that these two phenotypes may represent distinct developmental stages of a unified dormancy program governed by aggregate maturation (Pu et al., 2019; Bollen et al., 2021; Dewachter et al., 2021). During this progression, initially fluid liquid condensates accumulate extensive intermolecular β-sheet structures and undergo liquid-to-solid phase separation. This phase shift is proposed to increase disaggregation resistance, impair cellular resuscitation capacity, and ultimately facilitate the shift toward profound dormancy (Bollen et al., 2025).

Furthermore, specific molecular cascades can accelerate this phase-transition cascade. For instance, expression of the inner membrane toxin TisB in *E. coli* induces severe ATP exhaustion and stimulates protein aggregation, which in turn triggers systemic physiological arrest to sustain deep dormancy. Meanwhile, this toxin increases the rate-limiting barrier of chaperone-mediated disaggregation and substantially prolongs resuscitation lag time, constituting a mechanism coordinating stress resistance during dormancy and the temporal kinetics of resuscitation (Leinberger et al., 2024). Similarly, deletion of endogenous phage shock protein A (PspA) decelerates global metabolism, depletes intracellular ATP pools and accelerates aggregate buildup, thereby exacerbating dormancy and extending lag phases prior to regrowth. Conversely, supplementation with exogenous glucose restores cellular ATP concentrations and effectively reverses the persister phenotype (Li et al., 2024).

Accordingly, a broadly held notion is that reduced intracellular ATP availability acts both as a critical accelerator of protein aggregation and a quantitative biomarker for dormancy depth. The length of lag phase required for bacteria to exit dormancy and resume proliferation is directly determined by the ATP-fueled disaggregation capacity of the DnaK-ClpB chaperone system (Pu et al., 2019). Dynamic stable isotope labeling (SILAC) experiments reveal that persisters retain low basal levels of protein synthesis throughout dormancy and selectively enrich core components of DnaK, ClpB and the Clp protease machinery. At the early resuscitation stage, cells rapidly reactivate glycolytic and translational machineries while robustly synthesizing chaperones and protease complexes dedicated to aggregate disassembly and protein degradation (Semanjski et al., 2021). Hence, the successful awakening and full recovery of dormant bacteria are proposed to rely strictly on the ability of the intracellular chaperone network to completely disassemble mature aggregates and re-establish proteostasis once stress subsides (Stojowska-Swêdrzyńska et al., 2026). The recently proposed “proteostasis clock” framework posits that the dynamic assembly and disassembly of protein condensates precisely tune the duration of bacterial dormancy. Reversible macromolecular phase-transition kinetics not only define the magnitude of dormancy depth but also provide an integrated intrinsic temporal mechanism that explains how proteome status dictates the length of resuscitation lag following stress relief (Wang et al., 2026).

### 4.2 Irreversible protein aggregation-mediated cell death

Our understanding of bacterial aging has evolved considerably over time. The classical paradigm long held that bacteria proliferating via binary fission such as *E. coli* generate two biochemically equivalent daughter cells and are therefore incapable of aging (Kirkwood, 1977; Partridge and Barton, 1993; Nyström, 2002). Nevertheless, single-cell microscopic tracking performed by Stewart et al. in 2005 overturned this consensus. Even in morphologically symmetric division, mother cells inheriting the old cell pole exhibited steadily declining proliferation rates and markedly elevated mortality, providing the first single-cell evidence of bacterial aging (Stewart et al., 2005). This model was subsequently challenged by Wang et al. who deployed microfluidic platforms to monitor cell lineages over hundreds of generations and observed stable growth rates in mother cells, casting doubt on the universality of bacterial senescence (Wang et al., 2010). It was not until 2011 that Rang et al. reanalyzed these conflicting datasets using population genetic models. Their work validated that two opposing physiological trajectories coexist within *E. coli* populations: progressive aging and vitality restoration, both driven by asymmetric damage partitioning. Lineages retaining old versus new poles converge toward distinct steady-state doubling times, furnishing robust corroboration for the existence of bacterial aging (Rang et al., 2011).

Subsequent mechanistic studies uncovered that the microphysical basis underlying heterogeneous single-cell aging hinges tightly upon asymmetric segregation of damaged protein aggregates during cell division (Lindner et al., 2008; Winkler et al., 2010). *E. coli* employs an energy-dependent active transport machinery to traffic protein aggregates specifically toward cellular poles, assembling large polar inclusion bodies. This spatial sequestration strategy not only mitigates global metabolic disruption exerted by cytotoxic macromolecules but also lays the groundwork for asymmetric aggregate partitioning during cytokinesis and subsequent resuscitation of progeny cells (Rokney et al., 2009). Under oxidative stress, proteins subjected to irreversible carbonylation that evade clearance by PQC systems including Lon proteases readily crosslink into high-molecular-weight cytotoxic aggregates, which act as primary triggers and sustainers of progressive senescence (Nyström, 2005). Single-cell fluorescent tracking confirms that such aggregates are selectively retained at the old pole of mother cells upon division, with polar localization governed predominantly by a passive physical exclusion mechanism termed nucleoid occlusion: combined effects of restricted molecular diffusion and macromolecular crowding within the nucleoid compartment (Winkler et al., 2010; Coquel et al., 2013). Furthermore, the magnitude of asymmetric damage segregation is not static but dynamically amplified with escalating environmental stress (Vedel et al., 2016). Under lethal stress, misfolded proteins are preferentially partitioned to old-pole mother cells, driving their progressive aging (Proenca et al., 2019; Linnik et al., 2024), whereas nascent daughter cells inherit reduced aggregate loads and recover full metabolic capacity (Lindner et al., 2008; Rang et al., 2011). This evolutionary trait confers enhanced adaptability and survival advantages at the population level (Chao, 2010; Baig et al., 2014b; Vedel et al., 2016).

Nonetheless, the deployment of high-resolution single-cell tracking techniques has challenged the simplistic unidirectional causal relationship between protein aggregation and senescence. Aggregates asymmetrically retained in mother cells following sublethal stress do not induce prominent growth defects; instead, these assemblies function as long-term epigenetic memory modules. By selectively enriching chaperone machineries, they augment cellular survival against subsequent lethal stressors (Govers et al., 2018). This finding implies that the physiological functions of aggregates are far more complex than the simplistic “toxic versus protective” binary framework. Meanwhile, under ideal culture conditions free of exogenous stress, old-pole mother cells manifest intrinsic aging marked by elongated division cycles without concomitant accumulation or asymmetric sorting of misfolded protein aggregates (Łapińska et al., 2019). This observation implies parallel aging pathways independent of protein aggregation. Even if the central role of aggregation in aging is acknowledged, the underlying mechanisms may be more complex than mere biochemical toxicity. Recent work further demonstrates that the core driver of physiological senescence in *E. coli* may not be the intrinsic biochemical toxicity of aggregates, but their occupied intracellular physical volume. Mother cell proliferation rates decline as a mathematical function of reduced available cytoplasmic space, suggesting that aggregation-mediated aging may fundamentally arise from size-dependent intracellular spatial competition (Wagener et al., 2025). These findings thus suggest that aggregation is unlikely to constitute a general precondition for the onset of bacterial cell aging.

Although asymmetric damage isolation alleviates population-wide decline at the macroscopic level (Lindner et al., 2008; Baig et al., 2014b), the interplay between protein aggregation and bacterial death proves far more intricate than its association with aging at the single-cell scale. Aging refers to progressive deterioration of reproductive and survival potential, whereas cell death constitutes an irreversible termination of all vital processes–these two phenomena represent distinct evolutionary outcomes (Ksiazek, 2010; Moger-Reischer and Lennon, 2019). Whether arising from chronic cumulative damage or acute environmental insult, protein aggregation surpassing the cellular compensatory threshold becomes a decisive trigger of lethality (Tyedmers et al., 2010; Winkler et al., 2010; Moger-Reischer and Lennon, 2019; Proenca et al., 2019). Experimental data demonstrate that intracellular aggregate abundance rises significantly with prolonged cultivation and correlates positively with dead cell density; dead cells contain over fivefold higher aggregate loads relative to viable counterparts. Even in initially fully viable populations, subpopulations bearing elevated aggregate burdens succumb preferentially upon stress exposure, establishing pre-existing aggregate load as a precise biomarker predictive of population survival fate (Maisonneuve et al., 2008). When bacteria encounter extreme oxidative stress or copper toxicity, extensively crosslinked cytotoxic aggregates rapidly deplete chaperone networks, provoking fatal proteotoxic stress culminating in cell death (Zuily et al., 2022; Cornejo et al., 2023). Destabilization of the native quaternary structure of essential endogenous proteins (e.g., dissociation of functional tetramers into free monomers) can likewise initiate lethal aggregation (Pollack et al., 2024).

Notably, this endogenous protective program of stress-induced aggregate formation stands in stark contrast to synthetic strategies that artificially trigger protein aggregation as broad-spectrum antibacterial agents. For instance, ectopic expression of aggregation-prone peptide P33 initiates co-translational aggregation of nascent polypeptides, inducing ribosomal stalling and collapsing global proteostasis. This intervention efficiently eradicates both planktonic and biofilm-embedded forms of multiple multidrug-resistant ESKAPE pathogens, offering an innovative therapeutic paradigm to circumvent classical antibiotic resistance barriers (Khodaparast et al., 2025).

## 5 Conclusions and future perspectives

Proteostasis is fundamental for maintaining bacterial physiology and ensuring cellular adaptation to fluctuating environments. When endogenous or exogenous stresses disrupt the balance among protein synthesis, folding, and degradation, misfolded proteins accumulate and assemble into aggregates (Tyedmers et al., 2010; Balchin et al., 2016; Schramm et al., 2020). For decades, protein aggregation was primarily regarded as a toxic byproduct of proteostasis failure, reflecting the accumulation of damaged proteins and progressive cellular deterioration (Taylor et al., 2002; Dobson, 2004; Morimoto, 2008; Bednarska et al., 2013). However, advances in single-cell tracking, high-resolution imaging, and quantitative proteomics have substantially reshaped this view. Increasing evidence suggests that protein aggregation is not merely a passive consequence of cellular damage but rather a dynamic biological process closely associated with bacterial adaptation, dormancy, aging, and cell fate transitions (Govers et al., 2018; Pu et al., 2019; Dewachter et al., 2021; Kuczyńska-Wiśnik et al., 2023; Pei et al., 2025).

The current conceptual model propose that protein aggregation may regulate bacterial cell states through transitions between distinct physical phases. When the capacity of protein quality control systems remains sufficient, stress-induced protein condensates generated through LLPS may serve as reversible reservoirs for damaged proteins, facilitate metabolic remodeling, and promote entry into low-energy states that enhance bacterial survival under nutrient limitation, oxidative stress, or antibiotic exposure (Jin et al., 2021; Saurabh et al., 2022; Kuczyńska-Wiśnik et al., 2023; Pei et al., 2025; Guan et al., 2026). However, when cellular damage exceeds the buffering capacity of molecular chaperones and proteolytic systems, condensates may undergo maturation toward more stable gel-like or solid-like aggregates, which are associated with reduced molecular exchange, impaired clearance, and progressive loss of cellular function (Alberti and Hyman, 2021; Bollen et al., 2025). Thus, protein aggregation has been proposed as a potential regulatory node linking adaptive dormancy and irreversible cellular damage.

This emerging framework has received increasing support from studies investigating bacterial persistence and antibiotic tolerance. Early studies in *E. coli* demonstrated that endogenous aggregate accumulation during stationary phase correlates with increased formation of persister cells, while chemical perturbation experiments further suggested that protein aggregation contributes to antibiotic-tolerant phenotypes (Leszczynska et al., 2013). More recent high-resolution dynamic analyses have extended this concept by proposing that persister cells and VBNC cells may represent different stages along a continuum of aggregation-associated dormancy (Pu et al., 2019; Dewachter et al., 2021; Bollen et al., 2025; Sniezek et al., 2025). A recent model proposes that protein–RNA condensates formed through LLPS possess considerable phase plasticity. Initial liquid-like condensates may support shallow dormancy and rapid recovery upon environmental improvement, whereas further transitions toward gel-like or solid-like states may restrict molecular mobility and limit essential cellular processes, thereby promoting deeper dormancy and delayed resuscitation (Yang et al., 2026). While this framework has gained considerable empirical support, its explanatory power should not be conflated with mechanistic proof.

Nevertheless, an important question remains unresolved: whether changes in the physical properties of protein condensates directly drive transitions in bacterial physiological states or merely accompany such physiological transitions. Although liquid-to-solid phase transitions have been increasingly proposed as a critical determinant of dormancy depth and cell fate, current evidence is largely based on correlations between aggregate properties and physiological outcomes. Direct experimental demonstration that phase transitions themselves causally determine bacterial survival, persistence, or death remains limited. Therefore, the concept of protein aggregation as a “cell fate switch” should currently be viewed as an attractive working model rather than an established mechanism.

Indeed, the relationship between protein aggregation and bacterial cell fate is likely more complex and bidirectional than previously appreciated. On one hand, protein aggregates may actively regulate cellular states by sequestering damaged proteins, reorganizing metabolic networks, or facilitating entry into protective dormant states (Pu et al., 2019; Bollen et al., 2021, 2025; Dewachter et al., 2021). On the other hand, physiological transitions such as persistence, dormancy, and aging are frequently accompanied by reduced translation rates, impaired protein degradation capacity, and extensive remodeling of proteostasis networks, all of which may independently promote protein aggregation (Shah et al., 2006; Wood et al., 2013; Amato et al., 2014; Balaban et al., 2019; Steiner, 2021; Orman et al., 2026). Thus, aggregation may function both as a driver of cellular adaptation and as a downstream consequence of prolonged stress and metabolic reprogramming, depending on aggregate properties, cellular context, and stress duration.

For instance, under environmental stresses such as nutrient deprivation, oxidative stress, or heat shock, increased levels of misfolded proteins may primarily reflect disruption of proteostasis rather than an actively programmed aggregation response (Kwiatkowska et al., 2008; Winter et al., 2008; Tyedmers et al., 2010; Baig et al., 2014a; Iyer et al., 2018; Katikaridis et al., 2021; Lv et al., 2023; Aldikacti et al., 2026). Future studies should therefore distinguish the specific contributions of different types of aggregates, including dynamic condensates, intermediate aggregation states, and irreversible solid-like structures, to bacterial survival and death.

A similar uncertainty exists in bacterial aging. During replicative aging, bacteria often exhibit progressive accumulation of aggregated proteins together with reduced growth capacity and declining physiological performance (Maisonneuve et al., 2008; Winkler et al., 2010). Indeed, because aggregate accumulation and aging-associated phenotypes typically occur simultaneously, it remains difficult to determine whether aggregation initiates aging or represents a consequence of long-term damage accumulation.

Beyond these conceptual uncertainties, major technical and conceptual challenges remain. First, the biophysical threshold governing the transition from protective dynamic condensates to potentially toxic solid-like aggregates remains poorly defined. Current approaches for characterizing LLPS and liquid-to-solid transitions in living bacteria, including chemical perturbation using agents such as 1,6-hexanediol and fluorescence recovery after photobleaching (FRAP), have inherent limitations (Jin et al., 2021; Bollen et al., 2025). These methods may perturb the intracellular environment, and the small size of bacterial cells together with limited fluorescence recovery often complicates discrimination between highly viscous liquid states and gel-like assemblies (Kroschwald et al., 2017; Alberti et al., 2019; Bollen et al., 2025). Future development of minimally invasive approaches, integrating super-resolution microscopy, *in situ* cryo-electron microscopy, and quantitative biophysical measurements, will be essential for resolving aggregate dynamics under physiological conditions (Chen et al., 2024; Zheng and Cai, 2024; Carrion and Davis, 2025; Turner et al., 2025). Second, establishing direct causal relationships between aggregate properties and cellular outcomes remains a central challenge. Approaches that enable precise manipulation of aggregate formation, maturation, or clearance will be critical for determining whether specific aggregation states actively control bacterial fate or simply reflect broader stress responses. Moreover, the ecological significance of protein aggregation remains largely unexplored. Whether and how aggregation influences bacterial evolution, biofilm development, and microbial community interactions in complex natural environments represents an important direction for future investigation.

A deeper understanding of how protein aggregation regulates bacterial cell fate may also provide new opportunities for antimicrobial development. Conventional antibiotics primarily target specific physiological processes and are often less effective against slow-growing or dormant populations (Tuomanen et al., 1986; Eng et al., 1991; Kapoor et al., 2017; Lee et al., 2018; Baran et al., 2023; Halawa et al., 2024). Strategies targeting proteostasis networks may therefore offer alternative approaches for combating persistent and multidrug-resistant pathogens (Jeong et al., 2026). For example, preventing protective aggregation could theoretically restrict bacterial entry into persistence or deep dormancy states and improve antibiotic efficacy (Leszczynska et al., 2013; Pu et al., 2019). Conversely, disrupting protein quality control mechanisms or promoting uncontrolled irreversible aggregation could represent a strategy to overcome bacterial stress tolerance (Khodaparast et al., 2018; Zuily et al., 2022; Cornejo et al., 2023; Pollack et al., 2024; Khodaparast et al., 2025). That said, these approaches remain largely conceptual and require extensive validation. Key challenges include achieving pathogen specificity, minimizing toxicity to host cells and beneficial microbiota, and anticipating bacterial evolutionary adaptation to such interventions.

Overall, protein aggregation exhibits a dual role throughout the bacterial life cycle. Uncontrolled aggregation may contribute to proteotoxic stress, cellular aging, and death, whereas tightly regulated aggregation may provide a reversible adaptive strategy that enables survival under unfavorable conditions. Future integration of advanced imaging technologies, quantitative proteomics, synthetic perturbation systems, and single-cell causal analyses will be essential to move this field beyond descriptive associations toward mechanistic understanding. Such efforts are expected to ultimately clarify how protein aggregation contributes to bacterial survival, persistence, and fate determination, while potentially revealing new therapeutic vulnerabilities within bacterial proteostasis networks.
